# Prevalence of diabetes, metabolic syndrome and metabolic abnormalities in schizophrenia over the course of the illness: a cross-sectional study

**DOI:** 10.1186/1745-0179-2-14

**Published:** 2006-06-27

**Authors:** M De Hert, R van Winkel, D Van Eyck, L Hanssens, M Wampers, A Scheen, J Peuskens

**Affiliations:** 1 University Psychiatric Center Katholieke Universiteit Leuven, Leuvense Steenweg 517, 3070 Kortenberg, Belgium; 2 Department of Epidemiology and Public Health, University Liege, Belgium; 3 Department of Diabetes and Metabolic Disorders, CHU Sart Tilman, University Liege, Belgium

## Abstract

**Background:**

Patients with schizophrenia are at high risk of developing metabolic abnormalities.

**Method:**

A prospective study focusing on metabolic disturbances in patients with schizophrenia, including an oral glucose tolerance test, is currently ongoing at our University Hospital and affiliate services. The prevalence of metabolic abnormalities at baseline was assessed in a cohort of 415 patients with schizophrenia. The sample was divided into 4 groups according to duration of illness: first-episode patients (<1.5 years), recent-onset patients (between 1.5 and 10 years), subchronic patients (between 10 and 20 years) and chronic patients (>20 years).

**Results:**

Metabolic abnormalities were already present in first-episode patients, and considerably increased with increasing duration of illness. When compared to the general population matched for age and gender, much higher rates of the metabolic syndrome (MetS) and diabetes were observed for patients with schizophrenia. For MetS, the increase over time was similar to that of the general population. In contrast, the difference in the prevalence of diabetes in patients with schizophrenia and the general population dramatically and linearly increased from 1.6% in the 15–25 age-band to 19.2% in the 55–65 age-band.

**Conclusion:**

Thus, the current data suggest that on the one hand metabolic abnormalities are an inherent part of schizophrenic illness, as they are already present in first-episode patients. On the other hand, however, our results suggest a direct effect of the illness and/or antipsychotic medication on their occurence. The data underscore the need for screening for metabolic abnormalities in patients diagnosed with schizophrenia, already starting from the onset of the illness.

## 1. Background

Metabolic abnormalities have consistently been identified as a part of schizophrenic illness [1-4], but with the introduction of second generation antipsychotics and their possible association with metabolic abnormalities [5-7], the interest in this topic has been renewed.

Many studies have since then provided convincing evidence for a high risk of diabetes and other glucose abnormalities [5,8-12], the metabolic syndrome[13,14], and mortality due to elevated cardiovascular risk in patients with schizophrenia [15-18].

These metabolic abnormalities are of major clinical concern, not only because of their direct, somatic effects on morbidity and mortality, but also because of their association with psychiatric outcome, such as a higher prevalence of psychotic and depressive symptoms[19], a lower functional outcome [20], a worse percieved physical health [19] and lower adherence to medication [21].

The reasons that underlie the high prevalence of these metabolic abnormalities are much debated, especially when considering the possible role of second-generation, 'atypical' antipsychotics in the occurence of these abnormalities. On the one hand, many studies have suggested a role of (certain) atypical antipsychotics in the occurrence of metabolic abnormalities, both case reports [22-26], cross-sectional or retrospective studies [27,28] as prospective studies [29,30]. On the other hand, other studies have provided evidence for an increased prevalence of central obesity [31] and glucose abnormalities such as impaired fasting glucose and insulin resistance [32] in drug-naïve first-episode patients, suggesting that metabolic abnormalities are an inherent part of schizophrenic illness.

Most probably, these two views represent a polarisation of clinical reality, as medical factors (weight, age,...), psychosocial factors (environmental stress, exercise, dietary habits,...), genetic factors (family history of diabetes, possible genetic overlap with psychiatric illness) and pharmacological factors (weight gain, medication-induced lipid and glucose abnormalities) are likely to interact in their influence on the occurrence of metabolic abnormalities, making it difficult to disentangle the influence of each individual risk factor.

Next to the interrelationship of the individual risk factors, the lack of insight into the development and course of these metabolic abnormalities makes an evaluation of the best possible strategy to assess and treat these abnormalities difficult. If metabolic abnormalities are already present in the first illness episode of psychotic patients and are an inherent part of psychiatric illness, would it be useful then to invest in prevention strategies? On the other hand, if metabolic abnormalities are caused by an interplay of many risk factors, could early detection and treatment of these risk factors result in a reduction of the prevalence of these metabolic abnormalities, and thus in a reduction of the excess mortality in patients with schizophrenia?

To address these issues, the current study aimed to gather some insight into the development and course of metabolic abnormalities in schizophrenia, by assessing the presence of metabolic abnormalities in 4 cohorts of patients with schizophrenia: a cohort of first-episode patients with a duration of illness (DOI) of less than 1.5 years, a cohort of recent-onset patients with a DOI of 1.5 to 10 years, a cohort of subchronic patients with a DOI of 10 to 20 years and a cohort of chronic patients with a DOI of more than 20 years. It was hypothesized that metabolic abnormalities would already be present in first-episode patients, but that their prevalence would increase with DOI, in a rate that would be higher than that of the general population matched for age and gender.

## 2. Methods

All consecutive patients with a DSM-IV diagnosis of schizophrenia (SCH) or schizoaffective (SA), both out- (26.3%) or inpatients, of the university psychiatric hospital St. Jozef (Kortenberg, Belgium) and its affiliate services, were asked to participate in an extensive screening and prospective follow-up study of metabolic parameters. The prospective inclusions started in November 2003. At baseline, patients received a full fasting laboratory screening (including a full lipid profile and measurement of glycated haemoglobin (HbA1c)), clinical measurements and an ECG. A 75 gr glucose load Oral Glucose Tolerance Test (OGTT) was performed in all patients. Patients were initiated on an overnight fast and were monitored during the OGTT. Insulin resistance was measured using fasting glucose and insulin concentrations with the HOMA method. All laboratory analyses were performed in the same laboratory.

The sample was divided into 4 groups according to duration of illness: first-episode patients (DOI<1.5 years), recent-onset patients (DOI<10 years), subchronic patients (DOI between 10 and 20 years) and chronic patients (DOI >20 years).

The presence of the MetS was assessed using the ATP-III criteria [33], the adapted ATP-III criteria (fasting glucose ≥ 100 mg/dl instead of 110 mg/dl) [34] and the recent IDF criteria (Table 1) [35]. For the diagnosis of diabetes and prediabetic abnormalities, the criteria of The American Diabetes Association were used (Impaired fasting glucose (IFG ≥ 100 mg/dl and Impaired Glucose Tolerance (IGT), glucose ≥ 140 mg/dl at 2 hours in the OGTT) [36].

**Table 1:** Definitions of the metabolic syndrome.

	ATP III*	ATP III A*	IDF**
Criteria:			
Waist (cm)	M >102, F >88	M >102, F >88	M ≥94, F ≥80 Obligatory criterion
BP***	≥ 130/85	≥ 130/85	≥ 130/85
HDL (mg/dl)	M <40, F <50	M <40, F <50	M <40, F <50
TG (≥ 150 mg/dl)	≥ 150	≥ 150	≥ 150
Glucose (mg/dl)****	≥ 110	≥ 100	≥ 100

*MetS if 3 of 5 criteria are met.

**MetS if additional 2 criteria are met (waist is obligatory).

***Or if treated with antihypertensive medication.

****Or if treated with insulin or hypoglycaemic medication.

Descriptive statistics were computed for the basic demographic and clinical variables as well as for the variables relevant for the evaluation of metabolic abnormalities. The influence of the presence/absence of the metabolic syndrome and the presence/absence of glucose abnormalities on continuous variables was assessed by means of an independent samples t-test. The association between categorical variables was evaluated by a chi-square test.

In a second approach, in order to allow a comparison with the general population, the sample was dived into ten-year age-bands (15–25; 25–35; 35–45; 45–55 and 55–65 year old patients). The population data for the comparison for MetS came from the Asklepios Study [38]. In this study cardiovascular risk factors were evaluated by a primary care physician in 2,524 healthy subjects between 35 and 55. The data for the comparison of the prevalence of diabetes was obtained from an online Belgian Government report [39]. This report is based on all epidemiological data on diabetes available in Belgium and an evaluation of sales of antidiabetic medication, all ages, and a survey of pharmacies. A weighted mean was calculated for the general population age-bands to control for gender differences (percentage of male patients in the current study sample times prevalence of diabetes/MetS for male subjects from the general population, plus percentage of female patients in the current study sample times prevalence of diabetes/MetS for female subjects from the general population). This weighted mean for the general population was then compared to the mean of the study sample per age band.

The study was approved by an ethical committee and all patients gave written informed consent.

## 3. Results

### 3.1. Subjects

The mean age of the patients was 37.7 years (std 11.3) and the mean duration of illness was 10.8 years (std 10.2). Of all patients, 67.2% of patients were male; 99% were white and Belgian natives. The studied population consisted of 415 patients with schizophrenia: 100 first-episode patients (24.1%), 130 recent-onset patients (31.3%), 106 subchronic patients (25.5%) and 79 chronic patients (19.0%). The demographic characteristics of the different cohorts are shown in Table 2.

**Table 2:** Clinical and demographic data.

	FE	<10 yr	10 to 20 yr	>20 yr	p
Age	25.7 (± 8.2)	29.0 (± 7.5)	39.0 (± 6.1)	49.8 (± 5.8)	0.0001
Sex					ns
Female	26% (26)	33.1% (43)	40.0% (36)	39.2% (26)	
Male	74% (74)	66.9% (87)	66.0% (70)	60.8% (74)	
GAF	58.4 (± 10.8)	61.9 (± 7.5)	61.7 (± 9.0)	59.7 (± 8.9)	
Age first admission	25.2 (± 8.2)	23.8 (± 7.1)	24.2 (± 5.7)	22.2 (± 4.1)	0.0277
N admissions	1.6 (± 1.2)	3.9 (± 2.2)	6.8 (± 4.0)	9.0 (± 6.1)	0.0001
Duration illness	0.5 (± 0.4)	5.2 (± 2.4)	14.8 (± 2.8)	27.6 (± 5.7)	0.0001
N pills	2.1 (± 1.0)	2.8 (± 1.4)	3.8 (± 2.4)	4.2 (± 2.2)	0.0001
BMI	23.7 (± 1.3)	25.9 (± 5.2)	27.2 (± 5.3)	26.6 (± 5.0)	0.0001
BMI segmentation					0.0001
Normal	68% (68)	44.6% (58)	38.7% (41)	36.7% (29)	
Overweight	23% (23)	40.0% (52)	32.1% (34)	41.8% (33)	
Obese	9% (9)	15.4% (20)	29.2% (31)	21.5% (20)	
Living situation					0.0001
Sheltered housing	1% (1)	11.5% (15)	20.8% (22)	24.0% (19)	
With family	48% (48)	32.3% (42)	14.1% (15)	12.7% (10)	
Partner	14% (14)	7.7% ()10	7.6% (8)	15.2% (12)	
Alone	31% (31)	26.9% (35)	26.4% (28)	17.7% (14)	
Residential facility	6% (6)	21.6% (28)	31.1% (33)	30.4% (24)	
Occupation					0.0001
Work	11% (11)	3.0% (4)	6.6% (7)	7.6% (6)	
Sheltered work	0% (0)	0.8% (1)	4.7% ()5	1.3% (1)	
Study/training	25% (25)	15.4% (20)	10.4% (11)	1.3% (1)	
None	64% (64)	80.8% (105)	78.3% (83)	89.9% (71)	
Family history CVD	45% (45)	42.3% (55)	51.8% (55)	53.2% (45)	ns
Family history Diabetes	32% (32)	23.1% (30)	35.8% (38)	36.7% (29)	ns
Family history lipid disorder	34% (34)	36.1% (47)	36.8% (39)	30.4% (24)	ns

On average, patients took 3.2 (std 2.0) different medications. Antipsychotics were combined with anticholinergics (16%), antidepressants (38%), benzodiazepines (36%), mood stabilisers (21%) and somatic medication (41%) (Table 3). Regarding somatic medication, 0.7% (3 patients) were being treated with metformin, 1.7% of patients took a statin (7 patients) and 10.1% (42 patients) took antihypertensive medication. 68% of all patients were smokers, with no significant differences between groups.

**Table 3:** Medication in different groups.

	FE	<10 yr	10 to 20 yr	>20 yr	p
Anticholinergic	8% (8)	13.1% (17)	16.0% (17)	31.5% (25)	0.0002
Benzodiazepine	36% (36)	23.8% (31)	44.4% (46)	44.3% (35)	0.0003
Antidepressant	23% (23)	42.3% (55)	44.3% (47)	43.0% (34)	0.0043
Mood stabiliser	9% (9)	20.8% (27)	30.2% (72)	25.3% (20)	0.0019
					
Antipsychotic					0.0001
Only first generation	3 %(3)	4.6 %(6)	9.5% (10)	20.5% (16)	
Only second generation	94% (94)	87.7% (113)	74.5% (79)	60.3% (47)	
Combination	3% (3)	7.6% (10)	16.0% (17)	19.2% (15)	
					
Second generation AP	97% (97)	94.6% (123)	90.5% (96)	78.5% (62)	
Second generation (N = 400 prescriptions)					0.0001
Amisulpride (n = 32)	5% (5)	5.8% (6)	16.0% (17)	5.9% (4)	
Aripiprazole (n = 4)	3% (3)	0.7% (1)	0% (0)	0% (0)	
Clozapine (n = 74)	3% (3)	15.5% (20)	29.1% (30)	30.9% (21)	
Risperidone (n = 98)	32% (32)	20.2% (26)	23.3% (24)	23.5% (16)	
Quetiapine (n = 53)	10% (10)	10.7% (25)	10.7% (11)	10.3% (7)	
Olanzapine (n = 139)	47% (47)	31.1% (32)	31.1% (32)	29.4% (20)	

All but 2 patients were treated with antipsychotic medication at the time of assessment. First-generation antipsychotics were used by 19.3% (n = 80) of patients, second-generation antipsychotics by 91.1% (n = 378). The majority of patients were treated with one antipsychotic (n = 349, or 84.1%); 90.0% of this group received a second-generation antipsychotic, 10.0% a first-generation antipsychotic. Patients in the first-episode group were more likely to receive second-generation antipsychotics, to take a smaller number of different medications and to be on monotherapy (Table 3).

### 3.2. Metabolic abnormalities according to duration of illness

First-episode patients had a normal BMI and BMI segmentation (normal, overweight and obese). With increasing DOI, weight also significantly increased. Similarly, there was a significant increase in waist circumference (Tables 2 and 4). In all groups, there was a high prevalence of family history of both metabolic (diabetes and lipid abnormalities) as well as cardio-vascular disorders.

**Table 4:** Metabolic syndrome and criteria prevalence.

	FE	<10 yr	10 to 20 yr	>20 yr	p
MS ATP-III	17% (17)	21.5% (28)	34.9% (37)	36.7% (29)	0.0026
Criteria:					
Waist (M>102, F>88)	18% (18)	32.3% (42)	45.3% (48)	44.3% (42)	0.0001
BP (≥ 130/85)	43% (43)	34.6% (45)	57.5% (61)	64.7% (51)	0.0001
HDL (M<40 mg/dl, F<50 mg/dl)	26% (26)	27.7% (36)	31.1% (33)	31.6% (26)	ns
TG (≥ 150 mg/dl)	33% (33)	36.1% (47)	50.9% (54)	46.8% (37)	0.0252
Glucose (≥ 110 mg/dl)	3% (3)	2.3% (3)	11.3% (12)	20.2% (16)	0.0001
					
MS ATP-III A (AHA)	18% (18)	24.6% (32)	39.6% (42)	44.3% (35)	0.0001
Criteria:					
Waist (M>102, F>88)	18% (18)	32.3% (42)	45.3% (48)	44.3% (42)	0.0001
BP (≥ 130/85)	43% (43)	34.6% (45)	57.5% (61)	64.7% (51)	0.0001
HDL (M<40 mg/dl, F<50 mg/dl)	26% (26)	27.7% (36)	31.1% (33)	31.6% (26)	ns
TG (≥ 150 mg/dl)	33% (33)	36.1% (47)	50.9% (54)	46.8% (37)	0.0252
Glucose(≥ 100 mg/dl)	8% (8)	16.9% (22)	27.4% (29)	40.5% (32)	0.0001
					
MS IDF	17% (17)	28.5% (37)	42.4% (45)	49.4% (39)	0.0001
Criteria:					
Waist (M ≥ 94, F ≥ 80)	38% (38)	55.4% (72)	73.6% (78)	70.9% (56)	0.0001
BP (≥ 130/85)	43% (43)	34.6% (45)	57.5% (61)	64.7% (51)	0.0001
HDL (M<40 mg/dl, F<50 mg/dl)	26% (26)	27.7% (36)	31.1% (33)	31.6% (26)	ns
TG (≥ 150 mg/dl)	33% (33)	36.1% (47)	50.9% (54)	46.8% (37)	0.0252
Glucose (≥ 100 mg/dl)	8% (8)	16.9% (22)	27.4% (29)	40.5% (32)	0.0001

MetS was prevalent in all groups but significantly increased with increasing DOI, as did the individual criteria, except for low HDL (Table 4 and Figure 2). The frequency of elevated waist and glucose abnormalities was more than doubled in patients with a duration of illness of more than 10 years, when compared to first-episode patients. Female compared to male patients were significantly more often overweight or obese and more frequently met the waist circumference criterium, according to both ATP III and IDF criteria (*df *1, χ2 = 30.6 and χ2 = 16.36, *p *< .0.0001).

**Figure 1:**
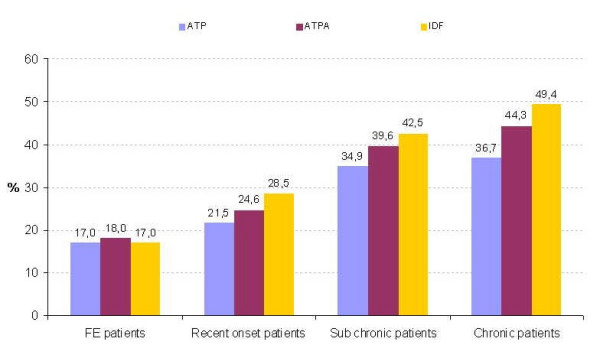
Prevalence of the metabolic syndrome per patient group. First-episode patients: duration of illness <1.5 years; recent-onset patients: duration of illness >1.5 years and <10 years; subchronic patients: duration of illness >10 years and <20 years; chronic patients: duration of illness >20 years.

**Figure 2:**
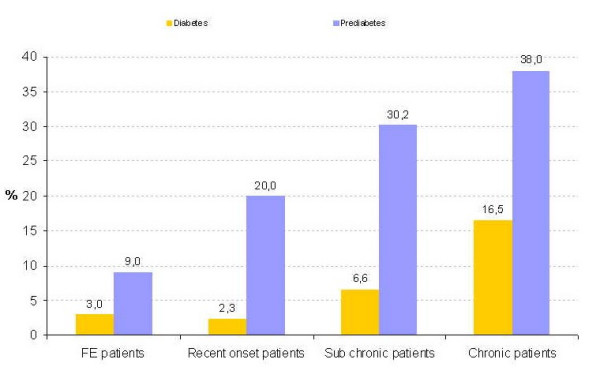
Glucose abnormalities per patient group. First-episode patients: duration of illness <1.5 years; recent-onset patients: duration of illness >1.5 years and <10 years; subchronic patients: duration of illness >10 years and <20 years; chronic patients: duration of illness >20 years.

In the total sample, 6.3% (n = 26) met criteria for diabetes, another 23.4% (n = 97) had prediabetic abnormalities, defined as impaired fasting glucose (IFG; fasting glucose >125 mg/dl) and/or impaired glucose tolerance (IGT; glucose ≥ 200 mg/dl at 120 minutes in the OGTT). Of the patients meeting criteria for diabetes, 12 (46.2%) met criteria with fasting values, 14 (53.8%) met criteria at 120 min in the OGTT and 4 (15.4%) met criteria both fasting and at 120 min in the OGTT. This means that when the current sample would have only been screened with a fasting glucose assessment, as suggested by the American Psychiatric Association/American Diabetes Association 37, only 12 of the 26 diabetes cases (46.2%) would have been identified. The prevalence of glucose abnormalities differed significantly between the different patient cohorts. The prevalence of diabetes increased from 3% in the first-episode and recent-onset group to 16.5% in the chronic group (Table 5 and Figure 2.). The mean values of parameters evaluated in the OGTT did not differ significantly between groups. Diabetes and glucose abnormalities were more frequent in patients treated with clozapine (*df *10, χ2 = 29.17, p < .0012). The distribution of glucose abnormalities over antipsychotic treatment regimes is shown in table 6.

**Table 5:** Glucose abnormalities.

	FE	<10 yr	10 to 20 yr	>20 yr	p
All abnormalities	12% (12)	22.3% (29)	36.8% (37)	44.5% (43)	0.0001
IFG, IGT	9% (9)	20% (26)	30.2% (32)	38.0% (30)	
Diabetes	3% (3)	2.3% (3)	6.6% (7)	16.5% (13)	
					
Fasting abnormalities	8% (8)	16.9% (22)	27.3% (29)	40.5% (32)	0.0001
IFG	6% (6)	16.1% (21)	24.5% (26)	32.9% (26)	
Diabetes	2% (2)	0.8% (1)	2.8% (3)	7.6% (6)	
					
Abnormalities at 120 min in OGTT	7% (7)	9.2% (12)	22.6% (24)	35.4% (28)	0.0001
IGT	6% (6)	6.9% (9)	17.9% (19)	24.0% (19)	
Diabetes	1% (1)	2.3% (3)	4.7% (5)	11.4% (9)	

**Table 6:** Glucose abnormalities in relation to antipsychotic treatment.

	Diabetes (n = 26)	Prediabetes (n = 97)	Normal values (n = 292)
Only FGA (n = 35)	8.6% (3)	25.7% (9)	65.7% (23)
Combination FGA + SGA (n = 45)	2.2% (1)	28.9% (13)	68.9% (31)
Combination SGA (n = 19)	5.3% (1)	21.0% (4)	73.7% (14)
			
Only 1 SGA (n = 314)	6.7% (21)	22.6% (71)	70.7% (222)
Amisulpride (n = 26)	0% (0)	3.9% (1)	96.1% (25)
Aripiprazole (n = 3)	0% (0)	0% (0)	100% (3)
Clozapine (n = 54)	9.3% (5)	42.6% (23)	48.1% (26)
Risperidone (n = 75)	6.6% (5)	22.7% (17)	70.7% (53)
Quetiapine (n = 44)	11.4% (5)	9.1% (4)	79.5% (35)
Olanzapine (n = 112)	5.4% (6)	23.2% (26)	71.4% (80)

As expected, the prevalence of the metabolic syndrome, regardless of the definition used, was significantly higher in diabetic subjects (ATP-III 76.7%, ATP-III A 80.0%, IDF 80.0%) compared to patients with prediabetic abnormalities (ATP-III 35.8%, ATP-III A 49.5%, IDF 54.1%) and patients without glucose abnormalities (ATP-III 17.5%, ATP-III A 17.7%, IDF 21.9%). Patients with MetS were more likely to meet criteria for diabetes or prediabetic abnormalities in all definitions of the MetS applied.

All parameters evaluated in the OGTT (glucose and insulin values fasting, at 30 minutes, at 60 minutes and 120 minutes) as well as HOMA-IR and glycated haemoglobin A1c were significantly different between patients with or without the metabolic syndrome regardless of the definition (p < .0001), with higher values in patients with MetS. Similar highly significant differences were found on all fasting serum lipid values and calculated lipid risk factors for cardiovascular disease (cholesterol, triglycerides, HDL, LDL, CHOL/HDL and LDL/HDL) (p < .0001).

The mean lipid values were lower in the first-episode group, although not statistically significant, in contrast to the frequency of abnormal lipid values that differed significantly for total cholesterol, triglycerides, LDL, CHOL/HDL and LDL/HDL (Table 7).

**Table 7:** Abnormal lipid values.

	FE	<10 yr	10 to 20 yr	>20 yr	p
CHOL (≥ 190 mg/dl)	27% (27)	45.4% (59)	61.3% (65)	60.8% (48)	0.0001
TG (≥ 150 mg/dl)	33% (33)	36.1% (47)	50.9% (54)	46.8% (37)	0.0252
HDL (M<40 mg/dl; F<50 mg/dl)	26% (26)	27.7% (36)	31.1% (33)	31.6% (26)	ns
LDL (≥ 115 mg/dl)	28% (28)	45.4% (59)	54.7% (58)	53.2% (42)	0.0005
CHOL/HDL (≥ 4)	30% (30)	42.3% (55)	53.8% (57)	49.4% (39)	0.0043
LDL/HDL (≥ 3)	14% (14)	23.1% (30)	33.0% (35)	32.9% (26)	0.0050

### 3.3. Comparison to the general population

The division of the study sample into age-bands resulted in a group of 98 patients in the 15–25 age-band, 127 in the 25–35 age-band, 99 in the 35–45 age-band, 71 in the 45–55 age-band and 20 in the oldest age-band (55–65).

**Figure 3:**
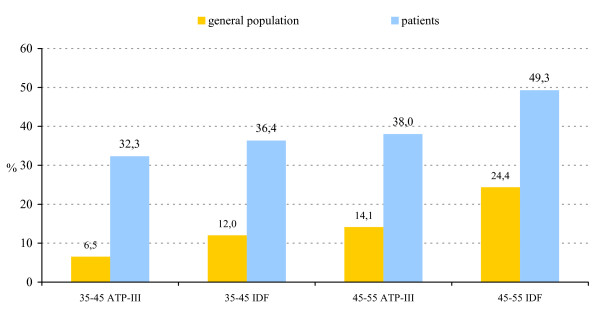
Prevalence of the metabolic syndrome per age-band in patients with schizophrenia compared to the general population according to ATP-III and IDF criteria.

Recent data on the prevalence of MetS according to ATP III and IDF criteria in the population were available for the age-bands 35–45 and 45–55 years old [38]. When compared to the calculated weighted mean prevalence for the general population, the prevalence of MetS was considerably higher in patients. The increase with age of the prevalence of MetS was similar in patients and the general population (Figure 3).

**Figure 4:**
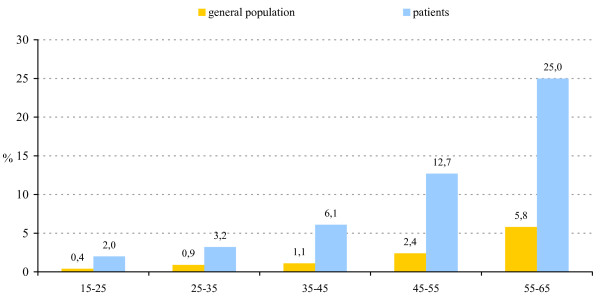
Prevalence of diabetes per age-band in patients with schizophrenia compared to the general population.

For diabetes, data were available on the prevalence for all the age-bands that were investigated in the present study [39]. In the age-band 15–25, a five times higher prevalence of diabetes was found when compared to the general population. With increasing age, the absolute difference between patients and the general population dramatically and linearly increased from 1.6% in the 15–25 age-band to 19.2% in the oldest age-band (Figure 4). The prevalence of diabetes per age-band, however, was 4 to 5 times higher in patients (15–25: 5.0; 25–35: 3.6; 35–45: 5.5; 45–55: 5.3 and 55–65: 4.3).

## 4. Discussion

### 4.1. Findings

The current study confirms the high prevalence of metabolic abnormalities in patients diagnosed with schizophrenia. In line with our hypothesis, metabolic abnormalities were already present in first-episode patients, but their prevalence considerably increased with increasing duration of illness. More specifically, the rise in the prevalence of metabolic abnormalities was most outspoken after an illness duration of ten or more years. When compared to the general population matched for age and gender, higher rates of the MetS and diabetes were observed for patients with schizophrenia. For the MetS, the increase of the prevalence was similar to that of the general population. In contrast, the difference in the prevalence of diabetes in patients with schizophrenia and the general population dramatically and linearly increased from 1.6% in the 15–25 age-band to 19.2% in the 55–65 age-band. The prevalence of diabetes per age-band was 4 to 5 times higher in patients with schizophrenia.

### 4.2. Prevalence of metabolic abnormalities and its implications for clinical practice

The data confirm that metabolic abnormalities are highly prevalent in a relatively young sample of schizophrenic patients treated with antipsychotics. Two large scale naturalistic studies in Belgium however revealed that the screening and diagnosis of these abnormalities in patients treated with antipsychotics still have not become routine practice, and that therefore, these abnormalities frequently remain untreated [40,41]. The data also suggest that when these patients would have been screened according to the current American Psychiatric Association/American Diabetes Association guidelines, relying only on fasting glucose, more than half of patients with diabetes (14 out of 26 or 53.8%) would not have been detected and thus, would not have been adequately treated. This is in line with the findings of Adam and Tarigan [42], and underscores the need for a more thorough screening in high-risk populations. Clearly, schizophrenic patients treated with antipsychotics ought to be considered at high risk of developing diabetes [43], as is confirmed by the current data.

Although considered costly and inconvenient by some [44,45], the use of OGTT's as a screening method for patients diagnosed with schizophrenia should therefore be encouraged, and certainly for very high risk patients presenting with IFG [46,47] or MetS. Moreover, the current data underscore the need for screening for metabolic abnormalities in patients diagnosed with schizophrenia, already starting from the onset of the illness, as suggested in the literature [12,47-49].

### 4.3. Evolution over time: expression of vulnerability or iatrogenic effect?

The current data suggest a greater vulnerability to develop metabolic abnormalities for patients diagnosed with schizophrenia when compared to the general population. A higher prevalence of metabolic abnormalities was already present in the age-band between 15 and 25 years old and increased with increasing age and duration of illness, which suggests a direct impact of the illness and/or negative metabolic side-effects of antipsychotic medication. The suggestion of a direct impact of the illness of schizophrenia and/or its pharmacological treatment on the development of metabolic abnormalities is in line with another Belgian study (BEST, i.e. "Belgian Evaluation of Screening and Treatment of high risk patients based on waist and age") [50]. This study was recently performed in 8,587 middle-aged (40–75 years) individuals without any cardiovascular history, consecutively selected by general practitioners upon a moderately increased waist circumference (≥ 80 cm in women and ≥ 94 cm in men). In this survey, 25 % of the non-diabetic population had the metabolic syndrome according to NCEP-ATP III criteria, thus a much lower prevalence than the 36.7 % observed in the subgroup of patients with the longest duration of the illness. This difference is striking since the subjects of BEST were older (61 vs 50 years) and had a much higher BMI (31.8 vs 26.6 kg/m^2^) when compared to the patients with chronic schizophrenia in our sample, obviously due to selection criteria. Furthermore, in the subgroup with the longest duration of illness, the prevalence of diabetes (16.5%) was almost similar to that observed in the non-psychiatric population of the BEST study (18%), despite a 10 year younger age and a 5 kg/m2 lower BMI. These findings support a deleterious metabolic effect of the illness of schizophrenia itself and/or antipsychotic medication.

Interestingly, the increase of the prevalence of the MetS with age was similar to that of the general population, meaning that although there was a higher prevalence of the MetS in patients with schizophrenia, the difference in prevalence with the general population remained more or less stable. In contrast, the difference in the prevalence of diabetes in patients with schizophrenia and the general population dramatically and linearly increased from 1.6% in the 15–25 age-band to 19.2% in the 55–65 age-band. The interpretation of these findings is difficult and needs to be done with caution, especially given the cross-sectional nature of the current study. They seem to suggest that the development of diabetes is not necessarily secondary to the development of MetS and that there could be an inherent vulnerability to diabetes in patients with schizophrenia, possibly aggravated by the metabolic side-effects of (some) antipsychotic medications. The finding that patients who were treated with clozapine were more likely to develop diabetes compared to patients treated with other antipsychotics is in line with this interpretation.

### 4.4. Methodological issues

This study also has some limitations. First and most importantly, it is a cross-sectional study. Therefore, one has to be cautious when interpreting the differences between patient groups, since cohort effects could (in part) explain the differences in the presence of metabolic abnormalities between patient groups. However, cohort effects are most likely to reduce differences between patients to the null in the present study, as the prevalence of diabetes and obesity has been rising rapidly in children, adolescents and young adults worldwide [51,52]. Second, this study was restricted to one site, indicating that the interpretation of the results needs to be done with caution, since large regional differences in metabolic parameters have been reported in the literature [53] Third, patients were not equally spread over the different age-bands, with especially a low number of patients in the oldest age-band (n = 20) which could have biased our findings in this specific age-band. Fourth, insufficient patients were included in the present study to allow a dichotomization according to gender over the different age-bands, although a significant influence of gender on the occurrence of metabolic abnormalities was found in the present study and was also reported in the literature [54, 55].

## 5. Conclusion

Metabolic abnormalities were already present in first-episode patients, but their prevalence considerably increased with increasing duration of illness. When compared to the general population matched for age and gender, higher rates of the MetS and diabetes were observed for patients with schizophrenia. For the MetS, the increase of the prevalence was similar to that of the general population. In contrast, the difference in the prevalence of diabetes in patients with schizophrenia and the general population dramatically and linearly increased from 1.6% in the 15–25 age-band to 19.2% in the 55–65 age-band. The prevalence of diabetes per age-band was 4 to 5 times higher in patients with schizophrenia. Thus, the current data suggest that on the one hand metabolic abnormalities are an inherent part of schizophrenic illness, as they are already present in first-episode patients. On the other hand, however, the current data suggest a direct effect of the illness and/or antipsychotic medication on the development of metabolic abnormalities, so that prevention strategies, identification of risk factors, early detection through adequate screening and treatment of metabolic abnormalities could well be of eminent importance in these patients. The current data underscore the need for screening for MetS, diabetes and lipid abnormalities in patients diagnosed with schizophrenia, already starting from the onset of the illness.

## Competing interests

The author(s) declare that they have no competing interests.

## Authors' contributions

Study planning and design: M. De Hert, L. Hanssens, A. Scheen, J. Peuskens

Data collection and statistical analysis: M. De Hert, R. van Winkel, D. Van Eyck, M. Wampers

Drafting report: all
